# Entropy-Based Stochastic Optimization of Multi-Energy Systems in Gas-to-Methanol Processes Subject to Modeling Uncertainties

**DOI:** 10.3390/e27010052

**Published:** 2025-01-09

**Authors:** Xueteng Wang, Jiandong Wang, Mengyao Wei, Yang Yue

**Affiliations:** 1 College of Electrical Engineering and Automation, Shandong University of Science and Technology, Qingdao 266590, China; wxtskd2@163.com (X.W.); mywei9701@163.com (M.W.); 2 Shandong Rongxin Group Co., Ltd., Zoucheng 273517, China; rxmhyy@163.com

**Keywords:** gas-to-methanol processes, multi-energy systems, entropy, stochastic optimization, modeling uncertainties

## Abstract

In gas-to-methanol processes, optimizing multi-energy systems is a critical challenge toward efficient energy allocation. This paper proposes an entropy-based stochastic optimization method for a multi-energy system in a gas-to-methanol process, aiming to achieve optimal allocation of gas, steam, and electricity to ensure executability under modeling uncertainties. First, mechanistic models are developed for major chemical equipments, including the desulfurization, steam boilers, air separation, and syngas compressors. Structural errors in these models under varying operating conditions result in noticeable model uncertainties. Second, Bayesian estimation theory and the Markov Chain Monte Carlo approach are employed to analyze the differences between historical data and model predictions under varying operating conditions, thereby quantifying modeling uncertainties. Finally, subject to constraints in the model uncertainties, equipment capacities, and energy balance, a multi-objective stochastic optimization model is formulated to minimize gas loss, steam loss, and operating costs. The entropy weight approach is then applied to filter the Pareto front solution set, selecting a final optimal solution with minimal subjectivity and preferences. Case studies using Aspen Hysys-based simulations show that optimization solutions considering model uncertainties outperform the counterparts from a standard deterministic optimization in terms of executability.

## 1. Introduction

The gas-to-methanol process is a chemical process primarily involving the purification and reforming of coke oven gas, which is followed by the synthesis and refining of methanol. During coal coking, significant amounts of coke oven gas are emitted directly into the atmosphere through combustion, causing serious environmental pollution and wasted energy. The gas-to-methanol process offers an effective way to repurpose this gas. In this process, the operating equipment includes various energy media such as gas, steam, and electricity [1,2]. Multi-energy systems are employed to optimize the allocation of these energies, thereby avoiding unnecessary energy losses associated with optimization of a single unit of energy [3].

Extensive research efforts have been devoted to optimizing multi-energy systems [4,5]. In real industries, numerous difficult-to-predict parameters, such as energy prices, energy demands, and ambient conditions, are commonly involved, making it essential to consider the impact of uncertainties [6,7,8,9]. Monte Carlo analysis [10], stochastic optimization [11], robust optimization [12], and modeling to generate alternatives [13] are four approaches for addressing such uncertainties. Among them, stochastic optimization may be the most commonly used approach in optimizing multi-energy systems. Rakipour et al. [14] built a combined energy system model, including electrical, heating, and cooling hubs to optimize profitability while accounting for uncertainties in demand and energy prices. Vahid-Pakdel et al. [15] optimized the operation of a multi-carrier energy system, implementing stochastic programming to address uncertainties in demand and market conditions. Song et al. [16] presented a coordinated optimization method for hydrogen-based integrated energy systems, addressing supply–demand mismatch through multi-time scale scheduling with storage and uncertainties. Xiao et al. [17] developed an optimization model for regional integrated energy systems incorporating uncertainties, multi-energy coupling, and demand response. Li et al. [18] explored a scheduling model for community-integrated energy systems using chance-constrained programming to minimize costs. Niknam et al. [19] formulated a stochastic model for optimal energy management in a grid-connected microgrid, using scenario-based programming and multi-objective optimization to minimize costs and emissions. Azizi et al. [20] introduced a mixed-integer linear programming model to minimize daily costs in a local multi-energy system using neural network forecasts and stochastic optimization. Mei et al. [21] proposed a stochastic optimization model for the optimal operation of integrated energy systems within an industrial park, addressing uncertainties in distributed power generation and multi-energy loads. Sakki et al. [22] obtained a versatile stochastic simulation-optimization framework for renewable energy systems for handling multiple uncertainties in system drivers and states. Lei et al. [23] conducted a multi-objective stochastic planning study for regional integrated energy systems, taking into account the uncertainties of renewable and multi-energy sources. In the aforementioned literature, modeling the uncertainties that have arisen from structural errors in mechanistic models gas not been considered [24,25,26].

The main contribution of this paper proposes an entropy-based stochastic optimization method for a multi-energy system in a gas-to-methanol process, aiming to achieve optimal allocation of gas, steam, and electricity to ensure executability under modeling uncertainties from structural errors. First, mechanistic models are developed for major equipments, including the desulfurization, steam boilers, air separation, and syngas compressors. Second, Bayesian estimation theory and the Markov Chain Monte Carlo approach are used to analyze the differences between historical data and model predictions, thereby quantifying modeling uncertainties. Third, subject to constraints in the model uncertainties, equipment capacities, and energy balance, a multi-objective stochastic optimization model is formulated to minimize gas loss, steam loss, and operating costs. The entropy weight approach is then applied to filter the Pareto front solution set, selecting a final optimal solution with minimal subjectivity and preferences.

In many industrial processes, modeling uncertainties plays a crucial role in achieving optimal energy allocation. Ignoring these uncertainties significantly reduces the executability of optimization solutions in practical applications. The main challenge is how to characterize the modeling uncertainties that arise from structural errors in mechanistic models and effectively integrate them into multi-energy optimization. To the best of our knowledge, modeling uncertainties from structural errors is seldom considered in the literature, and the proposed method resolves the above-mentioned challenge. To simplify the optimization problem, the impacts of other uncertainties, such as energy prices, energy demands, and ambient conditions, are neglected. These uncertainties have been extensively discussed in the existing literature. Should there be a need to consider their effects, the conclusions in the above literature can be consulted.

The rest of this paper is organized as follows: The problem to be solved is described in Section 2. Section 3 presents detailed steps of the proposed method. Case studies based on Aspen Hysys are provided in Section 4. Section 5 gives concluding remarks.

## 2. Problem Description

**Figure 1:**
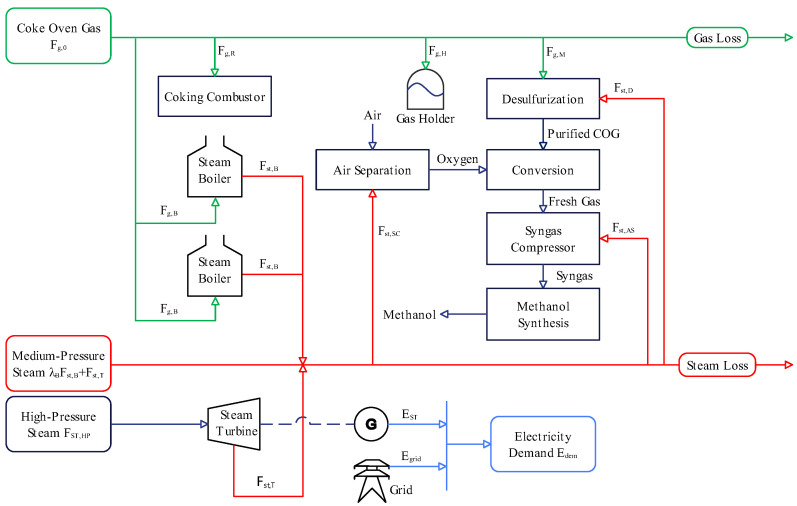
Schematic diagram of the multi-energy system in a gas-to-methanol process.

Figure 1 shows a schematic diagram of the multi-energy system in a gas-to-methanol process comprising a gas subsystem (green), a steam subsystem (red), and an electrical subsystem (blue). In this system, coke oven gas is the main by-product gas during the coal coking, and its supply ($F_{g,0}$) depends mainly on the coke production plan. Half of the gas ($F_{g,R}$) is returned to the coking combustor to provide heat for the coking chamber. The remaining portion of the gas is consumed in three parts: First, the gas flows into the methanol production line ($F_{g,M}$). This gas undergoes desulfurization to remove organic sulfur and then enters the conversion subprocess, where the ${\mathrm{CH}}_{4}$ and unsaturated hydrocarbons in the gas react with pure oxygen in oxidation and steam conversion reactions to produce the effective components needed for synthetic methanol, such as $H_{2}$, CO, and ${\mathrm{CO}}_{2}$. The syngas compressor then transports these components to the synthesis subprocess, where methanol synthesis occurs under the presence of a catalyst. Most of the gas separated after synthesis is recycled to replenish fresh gas, with the remaining portion sent to a subsidiary for hydrogen extraction. This process is specific in that the syngas is distinguished by a high hydrogen concentration (about 70%). As a result, the hydrogen addition requirement is not considered, and the expense associated with hydrogen recovery is excluded from operating costs [27,28]. Second, the gas ($F_{g,B}$) is burned in a steam boiler using the combustion heat to warm deaerated water and generate medium-pressure steam ($F_{st,B}$). Third, to ensure the safety and stability of the methanol production, the gas holder balances the surplus or shortage of gas in the system by regulating the storage and release of gas ($F_{g,H}$).

Steam is a crucial energy source for providing heat and power in this system, being classified into three pressure levels. High-pressure steam is purchased externally and is solely utilized by the steam turbine. The purchase quantity is determined based on the extraction of medium-pressure steam ($F_{st,T}$). Medium-pressure steam is supplied by the steam boiler and steam turbine, and it is exclusively used in three pieces of equipment: desulfurization ($F_{st,D}$), air separation ($F_{st,AS}$), and syngas compressor ($F_{st,SC}$). Their consumption is closely related to the gas flow rate $F_{g,M}$. Notably, high-pressure and medium-pressure steam are not consumed in the conversion and synthesis subprocesses. Low-pressure steam is supplied through the waste heat boilers and heat recovery boilers, and it is used for heat exchange in equipment such as deaerators and reboilers. The reaction heat released during the conversion and synthesis is recovered through the waste heat boilers and converted into low-pressure steam. Due to the relatively low utilization value of low-pressure steam, it is excluded from the energy scope to be considered in the sequel [28].

Electricity is supplied by both the steam turbine and the grid. The superheated steam discharged from the steam turbine drives a generator, which supplies electricity to the system. The electricity gap is balanced by purchasing electricity ($E_{grid}$) from the grid. The self-generated electricity ($E_{ST}$) is related to the coupling relationship between the steam flow rates $F_{st,T}$ and $F_{st,HP}$. The electricity demands of various equipment across the system, such as pumps and coolers, are collectively considered as a total electricity demand ($E_{dem}$), which is directly provided by the dispatchers.

Given data samples of the gas supply $F_{g,0}$, electricity demand $E_{dem}$, and initial volume of the gas holder $V_{H,0}$, the objective of this paper is to determine the optimal values of decision variables by minimizing gas loss, steam loss, and operating costs. Based on the above energy analysis, the decision variables include the extraction steam flow rate of the steam turbine ($F_{st,T}$), the gas flow rate of the methanol production line ($F_{g,M}$), the combustion gas flow rate of the steam boiler ($F_{g,B}$), and the gas throughput of the gas holder ($F_{g,H}$). In this optimization problem, gas loss refers to the coke oven gas venting, while steam loss refers to the excess medium-pressure steam produced. Operating costs require the following parameters to be specified: the coke oven gas purchase price ($c_{g}$), high-pressure steam purchase price ($c_{st,HP}$), electricity grid purchase price ($c_{grid}$), self-generated electricity price ($c_{ST}$), and medium-pressure steam selling price ($c_{st.MP}$). Optimization constraints arise from the equipment capacities, energy balance, and model uncertainties. Equipment capacities limit the upper and lower bounds of energy production or consumption for equipment such as the steam turbine, gas holder, and steam boiler. Energy balance requires that the supply of coke oven gas and medium-pressure steam be at least equal to the demand, while the supply of electricity must exactly match the demand. Model uncertainties are the ones resulting from structural errors in the mechanistic models of desulfurization, steam boilers, air separation, and syngas compressors under varying operating conditions. Taking the model uncertainties as constraints is critically important for achieving a satisfactory executability of optimization solutions (to be shown later in Section 4).

## 3. The Proposed Method

### 3.1. Mechanistic Modeling

This subsection presents mechanistic models for the desulfurization, steam boiler, air separation, and syngas compressor.

#### 3.1.1. Desulfurization Modeling

The steam consumption in the desulfurization is mainly influenced by the heat release of sulfur and the heat absorption of steam. The mathematical modeling of the desulfurization can be expressed as

(1) $$F_{st,D}=\frac{T_{su,out}-T_{su,in}}{T_{st,in}-T_{st,out}}\cdot \left(\xi_{D,1}\cdot {\left(F_{g,M}\right)}^{2}+\xi_{D,2}\cdot F_{g,M}+\xi_{D,3}\right),$$

where $\xi_{D,1}=\frac{C_{su}C_{D,1}}{C_{st}}$, $\xi_{D,2}=\frac{C_{su}C_{D,2}}{C_{st}}$, and $\xi_{D,3}=\frac{C_{su}C_{D,3}}{C_{st}}$ are unknown parameters of the mechanistic model. $C_{D,1}$, $C_{D,2}$, and $C_{D,3}$ are polynomial coefficients that characterize a coupling relationship between the gas flowrate and sulfur mass flow rate [29].

#### 3.1.2. Steam Boiler Modeling

The steam boiler converts the chemical energy of burning gas into thermal energy to generate steam. The steam generation flow rate of the steam boiler can be calculated as

(2) $$F_{st,B}=\frac{\left(100-\xi_{B,1}\cdot \left(1-\frac{\xi_{B,2}}{100}\right)\cdot \frac{{(T}_{EG}-T_{CA})}{100}-\xi_{B,2}\right)\xi_{B,3}F_{g,B}}{100\left(H_{S}-H_{FW}\right)},$$

where $\xi_{B,1}=k_{B,1}+k_{B,2}\cdot \alpha_{EG}$, $\xi_{B,2}=q_{L}$, and $\xi_{B,3}=Q_{B}$ are unknown parameters of the mechanistic model [30].

#### 3.1.3. Air Separation Modeling

The air separation is driven by a steam turbine that powers an air compressor and a booster. The steam consumption of the air separation is described as

(3) $$F_{st,AS}=\sum_{l=1}^{2}\frac{T_{AS,in,l}ln\frac{P_{AS,out,l}}{P_{AS,in,l}}\cdot \left(\xi_{AS,1}F_{g,M}+\xi_{AS,2}\right)}{\eta_{T}T_{T,in}\left(1-{\left(\frac{P_{T,out}}{P_{T,in}}\right)}^{\frac{\xi_{AS,3}-1}{\xi_{AS,3}}}\right)}.$$

Here, $\xi_{AS,1}=\sum_{l=1}^{2}\frac{R\rho_{A,l}\alpha_{O_{2}}}{\eta_{I,l}C_{st}}$, $\xi_{AS,2}=\sum_{l=1}^{2}\frac{R\rho_{A,l}\beta_{O_{2}}}{\eta_{I,l}C_{st}}$, and $\xi_{AS,3}=k_{T}$ are unknown parameters of the mechanistic model, $\alpha_{O_{2}}$ is a ratio of oxygen to coke oven gas in the converter, and $\beta_{O_{2}}$ is a correction factor [31].

#### 3.1.4. Syngas Compressor Modeling

The syngas compressor uses three-stage compression, and its modeling logic is consistent with the air separation. The mechanistic model for the *l*-th stage (*l* = 1, 2, 3) of the syngas compressor can be represented as

(4) $$F_{st,SCl}=\xi_{SCl,1}\frac{\frac{m_{l}}{m_{l}-1}T_{SCl,in}\left({\left(\frac{P_{SCl,out}}{P_{SCl,in}}\right)}^{\frac{m_{l}-1}{m_{l}}}-1\right)}{\eta_{Tl}T_{T,in}\left(1-{\left(\frac{P_{T,out}}{P_{T,in}}\right)}^{\frac{\xi_{SCl,2}-1}{\xi_{SCl,2}}}\right)}\cdot F_{l},$$

where

(5) $$\begin{array}{cc} \; & \;\left\{\begin{array}{cc} \; & m_{l}=\frac{\ln\left(\frac{P_{SCl,out}}{P_{SCl,in}}\right)}{\ln\left(\frac{T_{SCl,in}}{T_{SCl,out}}\right)+\ln\left(\frac{P_{SCl,out}}{P_{SCl,in}}\right)},\\ \; & F_{1}=F_{2}=\gamma_{f}\cdot F_{g,M},\\ \; & F_{3}=F_{2}+C_{SC,1}\cdot {\left(F_{g,M}\right)}^{2}+C_{SC,2}\cdot F_{g,M}+C_{SC,3},\\ \; & F_{st,SC}=F_{st,SC1}+F_{st,SC2}+F_{st,SC3}. \end{array}\right. \end{array}$$

Here, $\xi_{SCl,1}=\frac{{\rho_{l}R}_{u}}{M_{l}\eta_{P,l}C_{st}}$ and $\xi_{SCl,2}=k_{Tl}$ are unknown parameters of the mechanistic model, $F_{1}$ and $F_{2}$ represent the fresh gas coming from the conversion subprocess, and $\gamma_{f}$ is an empirical ratio of fresh gas to coke oven gas. $F_{3}$ is the syngas formed by the mixture of fresh gas and recycled gas, where $C_{SC,1}$, $C_{SC,2}$, and $C_{SC,3}$ are polynomial parameters unaffected by uncertainties. Equation (5) is the calculation formula for the polytropic exponent $m_{l}$ of the *l*-th stage of the syngas compressor [32].

#### 3.1.5. Methanol Synthesis Modeling

Methanol synthesis is conducted in a packed bed reactor. The chemical reactions include the ${\mathrm{CO}}_{2}$ hydrogenation reaction, CO hydrogenation reaction, and the reverse water-gas-shift (RWGS) reaction, as described in the following equations:

(6) $$CO_{2}+3H_{2}↔CH_{3}OH+H_{2}O\;\;\;\;\;\Delta H_{298}=-49.43\;\mathrm{kJ}/\mathrm{mol},$$

(7) $$CO+2H_{2}↔CH_{3}OH\;\;\;\;\;\;\;\;\;\;\;\;\;\;\;\Delta H_{298}=-90.55\;\mathrm{kJ}/\mathrm{mol},$$

(8) $$CO_{2}+H_{2}↔CO+H_{2}O\;\;\;\;\;\Delta H_{298}=+41.12\;\mathrm{kJ}/\mathrm{mol}.$$

Assuming ${\mathrm{CO}}_{2}$ as the primary source of carbon in methanol, the reaction rates for methanol synthesis in (6) and the RWGS reaction in (8) are calculated as [33]

(9) $$r_{MeOH}=\frac{k_{1}p_{{CO}_{2}}p_{H_{2}}\left(1-k_{6}\left(\frac{p_{H_{2}O}p_{MeOH}}{p_{H_{2}}^{3}p_{{CO}_{2}}}\right)\right)}{{\left(1+k_{2}\frac{p_{H_{2}O}}{p_{H_{2}}}+k_{3}\sqrt{p_{H_{2}}}+k_{4}p_{H_{2}O}\right)}^{3}},$$

(10) $$r_{RWGS}=\frac{k_{5}p_{{CO}_{2}}\left(1-k_{7}\left(\frac{p_{H_{2}O}p_{CO}}{p_{{CO}_{2}}p_{H_{2}}}\right)\right)}{1+k_{2}\frac{p_{H_{2}O}}{p_{H_{2}}}+k_{3}\sqrt{p_{H_{2}}}+k_{4}p_{H_{2}O}}.$$

Here, the syngas entering the reactor is assumed to behave as an ideal gas, with the fugacity $p_{co}$ of component co in the syngas being equivalent to its partial pressure, i.e., $p_{co}=n_{co}\cdot P_{PBR}$. The parameters of the steady-state kinetic models are based on the Arrhenius theory, namely, $k_{i}=A_{i}\cdot exp\left(\frac{B_{i}}{RT_{PBR}}\right)$, where i=1,2,3,4,5. The specific values of $A_{i}$ and $B_{i}$ are provided in Table 1. The equilibrium constants $k_{6}$ and $k_{7}$ are determined thermodynamically, with results provided by Graaf et al. [34] as

$${log}_{10}\frac{1}{k_{6}}=\frac{3066}{T_{PBR}}-10.592,\;\;\;\;{log}_{10}\frac{1}{k_{7}}=-\frac{2073}{T_{PBR}}+2.029.$$

The molar balance relationship is derived from Equations (6) and (8):

(11) $$\begin{array}{cc} \; & \;\left\{\begin{array}{cc} \; & \frac{dF_{CO}}{dW}=r_{RWGS},\;\;\;\;\;\;\;\;\;\;\;\;\;\;\;\;\;\frac{dF_{H_{2}O}}{dW}=r_{MeOH}+r_{RWGS},\\ \; & \frac{dF_{{CO}_{2}}}{dW}=-r_{MeOH}-r_{RWGS},\;\;\;\;\;\frac{dF_{MeOH}}{dW}=r_{MeOH},\\ \; & \frac{dF_{H_{2}}}{dW}=-3\cdot r_{MeOH}-r_{RWGS},\;\;\;\frac{dF_{{CH}_{4}}}{dW}=0. \end{array}\right. \end{array}$$

The variation in temperature $T_{PBR}$ inside the reactor is analyzed based on the energy balance, while the variation in pressure $P_{PBR}$ is described using the Ergun equation, with the formulas defined as

(12) $$\frac{dT_{PBR}}{dW}=\frac{-r_{MeOH}\Delta H_{MeOH}+r_{RWGS}\Delta H_{RWGS}}{\sum_{co}F_{co}C_{co}},$$

(13) $$\frac{dP_{PBR}}{dW}=-\frac{F_{PRB}}{{A_{c}}^{2}\rho_{c}D_{p}\emptyset^{3}}\left(\frac{150\left(1-\emptyset \right)\mu }{D_{p}}+\frac{1.75F_{PRB}\rho_{0}}{A_{c}}\right)\frac{P_{0}}{P_{PRB}}\frac{F_{PRB}}{F_{3}}\frac{T_{PRB}}{T_{0}}.$$

Here, the enthalpy changes ($\Delta H_{MeOH}$ and $\Delta H_{RWGS}$) for (6) and (8) are calculated as

$$\Delta H_{MeOH}=\Delta H_{298(6)}+\left(C_{MeOH}+C_{H_{2}O}-3C_{H_{2}}-C_{CO_{2}}\right)\cdot \left(T_{PBR}-298\right),$$

$$\Delta H_{RWGS}=\Delta H_{298(9)}+\left(C_{CO}+C_{H_{2}O}-C_{H_{2}}-C_{CO_{2}}\right)\cdot \left(T_{PBR}-298\right).$$

The heat capacity $C_{co}$ of component co at 298 K is represented as a polynomial function of $T_{PBR}$, with relevant thermodynamic parameters provided in [35]. The gas density $\rho_{0}$ is derived from the ideal gas law as

$$\rho_{0}=\frac{P_{PBR}\sum_{co}n_{co}M_{co}}{RT_{PBR}},$$

where $n_{co}$ and $M_{co}$ are the molar fraction and molar mass of component co, respectively.

#### 3.1.6. Identification of Unknown Parameters

The mechanistic models in Equations (1)–(5) can be rewritten in the following form:

(14) $$F_{st,x}=f_{x}\left(F_{g,x},\xi_{x},O_{x}\right),$$

$$F_{g,x}=\left\{\begin{array}{cc} F_{g,B}, & \mathrm{if}\;x=B,\\ F_{g,M}, & \mathrm{otherwise}. \end{array}\right.$$

Here, *x* is the subscript index for the steam boiler model (*B*), desulfurization model (*D*), air separation model (AS), and *l*-th stage of syngas compressor model (SCl). $F_{g,x}$ is the input variable of the model *x*, $F_{st,x}$ is the output variable of the model *x*, and $\xi_{x}=\left[\xi_{x,k};k=1,2,\ldots ,K_{x}\right]$ are unknown parameters of the model *x*, where $K_{x}$ denotes the number of unknown parameters in the model *x*. $O_{x}$ is the operating condition vector that comprises multiple state variables, with reference ranges and nominal values presented in Table 2. The other variables in the mechanistic models have fixed values shown in Table 3 [2,28].

The unknown parameters $\xi_{x}$ are estimated by minimizing the fitness between the measured data $F_{st,x}$ and the simulated data ${\hat{F}}_{st,x}=f_{x}\left(F_{g,x},\xi_{x},O_{x}\right)$ from the model in (14), which is defined as

(15) $$\xi_{x}=\underset{\xi_{x}}{\arg\min}\left(1-\sum_{i=1}^{I}\frac{{\left(F_{st,x}\left(i\right)-f_{x}\left(F_{g,x}\left(i\right),\xi_{x},O_{x}\right)\right)}^{2}}{{\left(F_{st,x}\left(i\right)-\frac{1}{I}\sum_{i=1}^{I}F_{st,x}\left(i\right)\right)}^{2}}\right),$$

where *I* is the number of discrete data points. Some existing optimization algorithms, such as the genetic algorithm, can be used to solve (15) to estimate $\xi_{x}$. The algorithm is a standard parameter estimation method. If outliers or noise are present in the historical data, existing methods for handling such issues will be employed [36]. For example, outliers will be treated as unknown parameters, while noise will be mitigated through data smoothing techniques (e.g., simple moving average) or noise filtering methods (e.g., wavelet denoising).

### 3.2. Uncertainty Quantification

Due to structural errors in the mechanistic models, the “current” model estimated using the optimization algorithm may not accurately describe the true behavior of chemical equipment under “future” operating conditions. To address this issue, this subsection employs Bayesian estimation theory and the Markov Chain Monte Carlo (MCMC) approach to quantify model uncertainties by analyzing the differences between historical data and model predictions under varying operating conditions.

#### 3.2.1. Bayesian Estimate

First, a prior parameter matrix $D_{x}^{\left(pr\right)}$ is acquired based on the $N_{x}^{\left(pr\right)}$ sets of historical data for model *x*. The *n*-th set of unknown parameters is estimated in (15) as $\xi_{x,n}^{\left(pr\right)}=\left[\xi_{x,nk}^{\left(pr\right)};k=1,2,\ldots ,K_{x}\right]$ by using the *n*-th set of historical data as the modeling data. After repeating the experiment $N_{x}^{\left(pr\right)}$ times, a prior parameter matrix $D_{x}^{\left(pr\right)}=\left\{\xi_{x,n}^{\left(pr\right)};n=1,2,\ldots ,N_{x}^{\left(pr\right)}\right\}$ can be obtained, which is defined as

(16) $$D_{x}^{\left(pr\right)}=\left[\begin{array}{ccc} \xi_{x,11}^{\left(pr\right)} & \cdots & \xi_{x,1K_{x}}^{\left(pr\right)}\\ \vdots & \ddots & \vdots \\ \xi_{x,N_{x}^{\left(pr\right)}1}^{\left(pr\right)} & \cdots & \xi_{x,N_{x}^{\left(pr\right)}K_{x}}^{\left(pr\right)} \end{array}\right].$$

Second, using Bayesian estimation theory with $D_{x}^{\left(pr\right)}$ as a condition, the posterior probability of the unknown parameters $\xi_{x}$ is estimated [37,38,39]. In the *k*-th dimension of $\xi_{x}$, the upper and lower bounds of the parameter $\xi_{x,k}$ are defined based on the maximum and minimum values of the corresponding parameter set ${\left\{\xi_{x,nk}^{\left(pr\right)}\right\}}_{n=1}^{N_{x}^{\left(pr\right)}}$ in $D_{x}^{\left(pr\right)}$. This enables the division of $\xi_{x,k}$ into $E_{k}$ equal intervals within that dimension. The interval division is uniformly applied across all $K_{x}$ dimensions of $\xi_{x}$, resulting in a total of *S* high-dimensional intervals, where $S=\prod_{k=1}^{K_{x}}E_{k}$. Assuming there are $N_{x,s}^{\left(pr\right)}$ sets of prior samples $\left[\xi_{x,1}^{\left(pr\right)},\xi_{x,2}^{\left(pr\right)},\cdots ,\xi_{x,K_{x}}^{\left(pr\right)}\right]$ in $D_{x}^{\left(pr\right)}$ that fall within the *s*-th interval, the probability of a sample within that interval is represented by a fictitious element $\theta_{x,s}$. Having determined the sequence of sample sizes $N_{x,s}^{\left(pr\right)}=N_{x,1}^{\left(pr\right)},N_{x,2}^{\left(pr\right)},\ldots ,N_{x,S}^{\left(pr\right)}$ within each interval, $\theta_{x,s}$ is expressed as

(17) $$\theta_{x,s}=\frac{N_{x,s}^{\left(pr\right)}}{N_{x}^{\left(pr\right)}}.$$

In the absence of any preference information, the prior distribution is assumed to follow a uniform distribution ($\theta_{x,s}∼U\left(0,1\right)$), i.e.,

$$f_{pr}\left(\theta_{x,s}\right)=\left\{\begin{array}{ll} 1, & {0\leq \theta }_{x,s}\leq 1,\\ 0, & \mathrm{otherwise}. \end{array}\right.$$

Let the candidate values for $\theta_{x,s}$ in $\left[0,1\right]$ be denoted as $\theta_{x,s}^{\left(1\right)},\theta_{x,s}^{\left(2\right)},\ldots ,\theta_{x,s}^{\left(J\right)}$. The prior probability $p\left(\theta_{x,s}\right)$ of $\theta_{x,s}$ can be formulated as

(18) $$p\left(\theta_{x,s}\right)=\frac{1}{J},$$

where *J* is the number of the candidate values. The likelihood probability $p\left(N_{x,s}^{\left(pr\right)}|\theta_{x,s}\right)$ of $\theta_{x,s}$ given $N_{x,s}^{\left(pr\right)}$ is calculated as

(19) $$p\left(N_{x,s}^{\left(pr\right)}|\theta_{x,s}\right)=\frac{N_{x}^{\left(pr\right)}!}{N_{x,s}^{\left(pr\right)}!\left(N_{x}^{\left(pr\right)}-N_{x,s}^{\left(pr\right)}\right)!}{\theta_{x,s}}^{N_{x,s}^{\left(pr\right)}}{\left(1-\theta_{x,s}\right)}^{N_{x}^{\left(pr\right)}-N_{x,s}^{\left(pr\right)}}.$$

Using Bayesian theory, combined with the prior and likelihood probability in Equations (18) and (19), the posterior probability of $\theta_{x,s}$ is represented by

(20) $$\begin{array}{ll} p\left(\theta_{x,s}|N_{x,s}^{\left(pr\right)}\right) & =\frac{p\left(\theta_{x,s},N_{x,s}^{\left(pr\right)}\right)}{\int p\left(\theta_{x,s},N_{x,s}^{\left(pr\right)}\right)d\theta_{x,s}},\\ & =\frac{p\left(N_{x,s}^{\left(pr\right)}|\theta_{x,s}\right)p\left(\theta_{x,s}\right)}{\sum_{j=1}^{J}p\left(N_{x,s}^{\left(pr\right)}|\theta_{x,s}^{\left(j\right)}\right)p\left(\theta_{x,s}^{\left(j\right)}\right)}. \end{array}$$

The conditional mean ${\bar{\theta }}_{x,s}^{\left(po\right)}$ is often considered as the posterior estimate of $\theta_{x,s}$, which is defined as

(21) $${\bar{\theta }}_{x,s}^{\left(po\right)}=E\left[\theta_{x,s}|N_{x,s}^{\left(pr\right)}\right]=\sum_{j=1}^{J}\theta_{x,s}^{\left(j\right)}\cdot p\left(\theta_{x,s}^{\left(j\right)}|N_{x,s}^{\left(pr\right)}\right).$$

Third, considering a specified sample size of $N_{x}^{\left(po\right)}$, ${\bar{\theta }}_{x,s}^{\left(po\right)}\cdot N_{x}^{\left(po\right)}$ sets of posterior samples $\left[\xi_{x,1}^{\left(po\right)},\xi_{x,2}^{\left(po\right)},\cdots ,\xi_{x,K_{x}}^{\left(po\right)}\right]$ are randomly generated from a uniform distribution within the *s*-th interval. The resulting posterior sample matrix within *S* intervals is

$$D_{x}^{\left(po\right)}=\left[\begin{array}{ccc} \xi_{x,11}^{\left(po\right)} & \cdots & \xi_{x,1K_{x}}^{\left(po\right)}\\ \vdots & \ddots & \vdots \\ \xi_{x,N_{x}^{\left(po\right)}1}^{\left(po\right)} & \cdots & \xi_{x,N_{x}^{\left(po\right)}K_{x}}^{\left(po\right)} \end{array}\right].$$

Kernel density estimation (KDE) is a nonparametric approach that estimates the overall PDF by smoothing each data point [40]. Based on the fundamental formula of KDE, the posterior PDF of $\xi_{x}$ can be described as

(22) $$f_{po}\left(\xi_{x}\right)=\frac{1}{N_{x}^{\left(po\right)}\prod_{k=1}^{K_{x}}h_{k}}\sum_{n_{1}=1}^{N_{x}^{\left(po\right)}}\prod_{k=1}^{K_{x}}K\left(\frac{\xi_{x,k}-\xi_{x,n_{1}k}^{\left(po\right)}}{h_{k}}\right).$$

Here, the bandwidth $h_{k}$ is the smoothing parameter of the *k*-th dimension of $D_{x}^{\left(po\right)}$. To avoid excessive smoothing of the nonunimodal distributions, Silverman’s rule of thumb is taken to calculate $h_{k}$ as

(23) $$h_{k}=0.9\cdot \min\left[\sigma_{k},\frac{{IQR}_{k}}{1.34}\right]\cdot {N_{x}^{\left(po\right)}}^{-\frac{1}{5}},$$

where $\sigma_{k}$ is the standard deviation of the *k*-th dimension of $D_{x}^{\left(po\right)}$, and ${IQR}_{k}$ is the interquartile range of the *k*-th dimension of $D_{x}^{\left(po\right)}$. The kernel function K(·) in (22) is chosen to be the Gaussian kernel, which is defined as

(24) $$K\left(u\right)=\frac{1}{\sqrt{2\pi }}e^{-\frac{u^{2}}{2}}.$$

#### 3.2.2. Markov Chain Monte Carlo Approach

The Metropolis–Hastings algorithm is employed to randomly sample from the high-dimensional posterior PDF of (22) in order to simulate the complete posterior distribution and estimate model parameters:

(1) Select the multivariate normal distribution as the proposal function q(u), and set the initial point of the Markov chain at m=0 as $\xi_{x,0}^{\left(mc\right)}=\frac{\sum_{n=1}^{N_{x}^{\left(pr\right)}}\xi_{x,n}^{\left(pr\right)}}{N_{x}^{\left(pr\right)}}$.
(2) Generate a new candidate parameter $\xi_{x,m^{*}}^{\left(mc\right)}$ from $q\left(\xi_{x,m^{*}}^{\left(mc\right)}|\xi_{x,m}^{\left(mc\right)}\right)∼N\left(\xi_{x,m}^{\left(mc\right)},\Sigma \right)$ based on the current parameter $\xi_{x,m}^{\left(mc\right)}$. The covariance matrix Σ is calculated from $D_{x}^{\left(pr\right)}$ as
  $$\Sigma =\frac{1}{N_{x}^{\left(pr\right)}-1}\sum_{n=1}^{N_{x}^{\left(pr\right)}}\left(\xi_{x,n}^{\left(pr\right)}-\xi_{x,0}^{\left(mc\right)}\right){\left(\xi_{x,n}^{\left(pr\right)}-\xi_{x,0}^{\left(mc\right)}\right)}^{T}.$$
(3) Calculate an acceptance probability α for $\xi_{x,m^{*}}^{\left(mc\right)}$ given $\xi_{x,m}^{\left(mc\right)}$, and generate a uniform random number β∼U(0,1). The acceptance probability α is defined as
  $$\alpha =\min\left[\frac{f_{po}\left(\xi_{x,m^{*}}^{\left(mc\right)}\right)}{f_{po}\left(\xi_{x,m}^{\left(mc\right)}\right)},1\right].$$
  If β≤α, accept $\xi_{x,m^{*}}^{\left(mc\right)}$ and set it as the next state, i.e., $\xi_{x,m+1}^{\left(mc\right)}=\xi_{x,m^{*}}^{\left(mc\right)}$. If β>α, reject $\xi_{x,m^{*}}^{\left(mc\right)}$ and keep the current state unchanged, i.e., $\xi_{x,m+1}^{\left(mc\right)}=\xi_{x,m}^{\left(mc\right)}$.

### 3.3. Optimization Modeling

This subsection first formulates the constraints and multi-objective functions of the multi-energy system in the gas-to-methanol process. Next, the entropy weight approach based on the Pareto front is provided.

#### 3.3.1. Optimization Constraints

In the multi-energy optimization problem, the energy balance constraints must be satisfied for each energy medium. The gas balance indictor ensures that the gas supply is greater than or equal to its consumption, as represented by

(25) $${F_{g,0}\geq F}_{g,R}+F_{g,M}+\lambda_{B}\cdot F_{g,B}+F_{g,H}+F_{g,H,L},$$

where

(26) $$F_{g,H,L}=\gamma_{H,L}\cdot V_{H},\;\;F_{g,R}=\gamma_{R}\cdot F_{g,0}.$$

Here, Equation (26) simulates the energy loss in the gas storage and the return gas needed to provide the heat required for coking. The steam consumption by the desulfurization, air separation, and syngas compressors must not exceed the steam generated from the steam boilers and steam turbines. The steam balance indicator can be expressed as

(27) $$F_{st,T}+\lambda_{B}\cdot f_{B}\left(F_{g,B},\xi_{B},O_{B}\right)\geq \sum_{\begin{array}{c} x=D,AS,SC1\\ ,SC2,SC3 \end{array}}f_{x}\left(F_{g,M},\xi_{x},O_{x}\right),$$

where $f_{D}$, $f_{AS}$, $f_{SC1}$, $f_{SC2}$, and $f_{SC3}$ correspond to the steam consumption models in (14), and $f_{B}$ corresponds to the steam generation model in (14).

The steam turbine expands high-pressure steam $F_{ST,HP}$ to medium-pressure steam $F_{ST,MP}$, converting the heat in the superheated steam into mechanical energy to drive the generator for electricity generation. Additionally, it extracts steam $F_{st,T}$ at the intermediate stage to satisfy industrial medium-pressure steam demand. The electricity generation of the steam turbine, defined as $E_{ST}$, can be described as

(28) $$E_{ST}=\frac{1}{3600}\eta_{ST}\cdot \sum_{r=MP,HP}F_{ST,r}H_{ST,r},$$

where the relationships of flowrates among $F_{ST,HP}$, $F_{ST,MP}$, and $F_{st,T}$ are expressed as

(29) $$F_{st,T}=\gamma_{ex}{\cdot F}_{ST,HP},\;\;F_{st,T}=F_{ST,HP}-F_{ST,MP}.$$

The electricity balance indicator, which equalizes the electricity generation amount with electricity demand, is represented as

(30) $$E_{ST}+E_{grid}=E_{dem}.$$

Based on process knowledge and practical engineering experience, the direction in which the system stores gas in the gas holder is defined as the positive direction. The gas holder volume reflects the imbalance between gas supply and consumption. When the gas holder operates within its designated operating range, it can effectively balance any surplus or shortage of gas in the system. The optimization constraints of the gas holder are described as follows:

(31) $$V_{H}=V_{H,0}+F_{g,H}-F_{g,H,L},$$

(32) $$V_{H}^{min}\leq V_{H}\leq V_{H}^{max},$$

(33) $$\left|V_{H}-V_{H,0}\right|\leq \min\left[V_{H}^{max}-V_{H,0},V_{H,0}-V_{H}^{min}\right].$$

Here, $V_{H,0}$ is the initial volume of the gas holder, and superscripts max and min are the maximum and minimum values of variables, respectively. The gas holder volume, adjusted by the optimization strategy in (31), must comply with the capacity constraint in (32), and the gas throughput should not exceed the limit in (33).

To maintain the normal operation of equipment, the production and consumption flowrates of primary energies like gas, steam, and electricity must operate within the following equipment capacity constraints:

(34) $$\left\{\begin{array}{ll} F_{g,0}^{min}\leq F_{g,0}\leq F_{g,0}^{max},\;\;F_{g,M}^{min}\leq F_{g,M}\leq F_{g,M}^{max}, & \\ F_{g,B}^{min}\leq F_{g,B}\leq F_{g,B}^{max},\;\;F_{g,H}^{min}\leq F_{g,H}\leq F_{g,H}^{max}, & \\ E_{ST}^{min}\leq E_{ST}\leq E_{ST}^{max},\;\;E_{dem}^{min}\leq E_{dem}\leq E_{dem}^{max}, & \\ F_{st,T}^{min}\leq F_{st,T}\leq F_{st,T}^{max},\;\;F_{st,B}^{min}\leq F_{st,B}\leq F_{st,B}^{max}, & \\ F_{st,AS}^{min}\leq F_{st,AS}\leq F_{st,AS}^{max},\;\;F_{st,SC}^{min}\leq F_{st,SC}\leq F_{st,SC}^{max}. & \end{array}\right.$$

Methanol synthesis does not involve the supply or consumption of coke oven gas or medium-pressure steam. And issues related to compression requirements, syngas flowrate, and electricity demand have been addressed in (4), (5), and (30). In addition, to ensure methanol production efficiency, the methanol flow rate $F_{MeOH}$ at the outlet of the synthesis reactor must exceed the minimum threshold set in the process operation manual, i.e.,

(35) $${\left(F_{MeOH}\right)}_{out}>F_{MeOH}^{min},$$

where ${\left(\cdot \right)}_{in}$ and ${\left(\cdot \right)}_{out}$ are the inlet and outlet of the reactor, respectively.

#### 3.3.2. Multi-Objective Functions

The optimization objective is to minimize the losses of gas and steam energy and operating costs under modeling uncertainties by reasonably allocating the three types of energy: gas, steam, and electricity. The multi-objective stochastic optimization model is constructed as

(36) $$\begin{array}{cc} \min_{x}f(x)= & {\left[f_{G}\left(x\right),f_{S}\left(x\right),f_{C}\left(x\right)\right]}^{T},\\ s.t.\;\;\mathrm{inequality}\;\mathrm{constraints}\; & \mathrm{in}\;(25),(27),(32),(33),(34),(35)\\ \;\;\;\;\;\;\;\;\mathrm{equational}\;\mathrm{constraints}\; & \mathrm{in}\;(1)–(5),(26),(31),(28)–(30).\\ \; \end{array}$$

Here, $x=\left(F_{st,T},F_{g,M},F_{g,B},F_{g,H}\right)$ are the decision variables of the optimization model. $f_{G}$ represents the energy loss function of gas:

(37) $$f_{G}=\left|{F_{g,0}-F}_{g,R}-F_{g,M}-\lambda_{B}\cdot F_{g,B}-F_{g,H}\right|,$$

where the gas loss is the difference between the supply and demand of gas in the system. Due to the difficulty in storing steam, to maintain the safety and stability of the equipment, some of the steam output from the steam boilers and steam turbines is not directly used for production. Therefore, the energy loss function $f_{S}$ of steam can be expressed as

(38) $$f_{S}=\left|\left(1-\gamma_{S}\right)\cdot \left(F_{st,T}+\lambda_{B}\cdot F_{st,B}\right)-F_{st,D}-F_{st,AS}-F_{st,SC}\right|.$$

In methanol production, high-pressure steam and coke oven gas serve as the primary fuels, and their associated purchase costs must be taken into account. Since the self-generated electricity from the steam turbine does not fulfill the entire electricity demand of the system, the cost of purchasing additional electricity from the grid must be factored in to achieve electricity balance. Furthermore, there are costs related to the self-generated electricity from the steam turbine. To maximize the utilization of surplus resources, this objective incorporates the sale of surplus medium-pressure steam into the operating costs. The objective function $f_{C}$ of the operating costs is

(39) $$\begin{array}{ll} f_{C}= & c_{st,HP}{\cdot F}_{ST,HP}+c_{g}{\cdot F}_{g,0}+c_{grid}\cdot E_{grid}+c_{ST}\cdot E_{ST}-\\ \; & c_{st,MP}\cdot \left(F_{st,T}+\lambda_{B}\cdot F_{st,B}-F_{st,D}-F_{st,AS}-F_{st,SC}\right). \end{array}$$

#### 3.3.3. Entropy Weight Approach Based on Pareto Front

Multi-objective optimization problems often involve multiple conflicting objective functions, and there is usually no single solution that can simultaneously optimize all objectives. The Pareto front represents the set of all possible nondominated solutions in multi-objective optimization [41]. Assuming there are *N* optimal solutions denoted as ${\left\{x_{p}\right\}}_{p=1}^{N}={\left\{\left(F_{st,T,p},F_{g,M,p},F_{g,B,p},F_{g,H,p}\right)\right\}}_{p=1}^{N}$, with three objective functions $f_{G}$, $f_{S}$, and $f_{C}$, the corresponding objective function values for ${\left\{x_{p}\right\}}_{p=1}^{N}$ can be represented as

(40) $$Y_{pq}=\left[\begin{array}{ccc} f_{G}\left(x_{1}\right) & f_{S}\left(x_{1}\right) & f_{C}\left(x_{1}\right)\\ \vdots & \vdots & \vdots \\ f_{G}\left(x_{N}\right) & f_{S}\left(x_{N}\right) & f_{C}\left(x_{N}\right) \end{array}\right].$$

If there does not exist a solution *x* such that $f_{q}\left(x\right)\leq f_{q}\left(x_{1}\right)$ for all objective functions (where q=1,2,3) and $f_{q^{*}}\left(x\right)<f_{q^{*}}\left(x_{1}\right)$ holds for at least one objective function (where $q^{*}\in q$), then $x_{1}$ belongs to the Pareto front.

In practical applications, optimization tasks often require a more specific solution. Therefore, the entropy weight approach is used to filter the ideal optimal solution on the Pareto front, effectively reducing the subjectivity and preferences among various objectives [42]. The proportion of $f_{q}\left(x_{p}\right)$ in $Y_{pq}$ is calculated by normalization, and the proportion matrix $P_{pq}$ of ${\left\{x_{p}\right\}}_{p=1}^{N}$ is expressed as

(41) $$P_{pq}=\left[\begin{array}{ccc} \frac{f_{G}\left(x_{1}\right)}{\sum_{p=1}^{N}f_{G}\left(x_{p}\right)} & \frac{f_{S}\left(x_{1}\right)}{\sum_{p=1}^{N}f_{S}\left(x_{p}\right)} & \frac{f_{C}\left(x_{1}\right)}{\sum_{p=1}^{N}f_{C}\left(x_{p}\right)}\\ \vdots & \vdots & \vdots \\ \frac{f_{G}\left(x_{N}\right)}{\sum_{p=1}^{N}f_{G}\left(x_{p}\right)} & \frac{f_{S}\left(x_{N}\right)}{\sum_{p=1}^{N}f_{S}\left(x_{p}\right)} & \frac{f_{C}\left(x_{N}\right)}{\sum_{p=1}^{N}f_{C}\left(x_{p}\right)} \end{array}\right].$$

In information theory, entropy is a measure of the uncertainty of a system or the uniformity of its distribution. According to the formula for information entropy, the entropy of $f_{q}$ is expressed as

(42) $$H_{q}=-\frac{1}{\ln N}\sum_{p=1}^{N}P_{pq}\ln P_{pq}.$$

When the information entropy of a specific objective is low, it indicates that the differences among the optimal solutions are small, reflecting a low degree of uncertainty for that objective. Therefore, the objective is assigned a higher weight. Conversely, a higher entropy value implies a more uniform distribution of solutions, with greater differences among them, leading to a lower weight for the objective. The entropy weight $w_{q}$ corresponding to $f_{q}$ is obtained as

(43) $$w_{q}=\frac{1-H_{q}}{\sum_{q=1}^{3}\left(1-H_{q}\right)}.$$

Considering the relative importance of various objectives in $Y_{pq}$ through ${\left\{w_{q}\right\}}_{q=1}^{3}$, the position $p^{*}$ of the ideal optimal solution on the Pareto front can be determined as

(44) $$p^{*}=\underset{p}{\arg\min}Y_{pq}\cdot \left[\begin{array}{c} w_{1}\\ w_{2}\\ w_{3} \end{array}\right].$$

### 3.4. Detailed Steps of the Proposed Method

**Figure 2:**
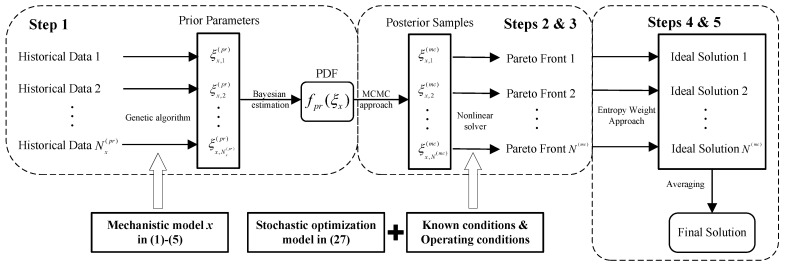
Flowchat of the proposed method.

As illustrated in Figure 2, the proposed method is outlined as the following steps:

(1) The mechanistic models for the desulfurization, steam boiler, air separation, and syngas compressor in (1)–(5) are first formulated, with model parameters to be estimated through the genetic algorithm. Next, the posterior PDFs of these parameters in (22) are fitted based on the Bayesian estimation theory, which are then employed to establish the multi-objective stochastic optimization model in (36).
(2) One set of parameter samples, denoted as $\xi_{D,1}^{\left(mc\right)}$, $\xi_{B,1}^{\left(mc\right)}$, $\xi_{AS,1}^{\left(mc\right)}$, $\xi_{SC1,1}^{\left(mc\right)}$, $\xi_{SC2,1}^{\left(mc\right)}$, and $\xi_{SC3,1}^{\left(mc\right)}$, is randomly drawn from the posterior PDFs using the MCMC approach in Section 3.2.2. These samples serve as inputs for the optimization model in (36), transforming the uncertain optimization problem into a deterministic one.
(3) The transformed problem is solved by a nonlinear solver, yielding a set of nondominated solutions on the Pareto front. As a typical nonlinear solver, the direct multi-search algorithm being implemented by the function “paretosearch” in Matlab is exploited here [43]. This solution set is evaluated by the entropy weight approach in (40)–(44), with the evaluation results being considered as the ideal optimal solution for the current samples, i.e., $\left(F_{st,T}^{*}\left(1\right),F_{g,M}^{*}\left(1\right),F_{g,B}^{*}\left(1\right),F_{g,H}^{*}\left(1\right)\right)$.
(4) The parameter samples are updated, and Steps 2 and 3 are repeated until the sampling criterion of $N^{\left(mc\right)}$ sets is met. Thus, the ideal optimal solutions across different parameter samples are obtained as ${\left\{F_{st,T}^{*}\left(m\right),F_{g,M}^{*}\left(m\right),F_{g,B}^{*}\left(m\right),F_{g,H}^{*}\left(m\right)\right\}}_{m=1}^{N^{\left(mc\right)}}$.
(5) The expected optimal values are adopted as the final solution for the optimization problem, being calculated as the mean of the $N^{\left(mc\right)}$ ideal optimal solutions:
  (45) $$\begin{array}{cc} \; & {\bar{F}}_{st,T}^{*}=\frac{\sum_{m=1}^{N^{\left(mc\right)}}F_{st,T}^{*}\left(m\right)}{N^{\left(mc\right)}},\;{\bar{F}}_{g,M}^{*}=\frac{\sum_{m=1}^{N^{\left(mc\right)}}F_{g,M}^{*}\left(m\right)}{N^{\left(mc\right)}},\\ \; & {\bar{F}}_{g,B}^{*}=\frac{\sum_{m=1}^{N^{\left(mc\right)}}F_{g,B}^{*}\left(m\right)}{N^{\left(mc\right)}},\;\;{\bar{F}}_{g,H}^{*}=\frac{\sum_{m=1}^{N^{\left(mc\right)}}F_{g,H}^{*}\left(m\right)}{N^{\left(mc\right)}}. \end{array}$$

Remark: In Step 1, alternative stochastic optimization approaches, such as sample average approximation, can also be employed [44]. In Step 3, other Pareto front decision-making approaches, such as the entropy-weight TOPSIS approach, can likewise be applied [45]. The solutions obtained using various approaches are provided in Section 4 for comparison.

## 4. Case Studies

**Figure 3:**
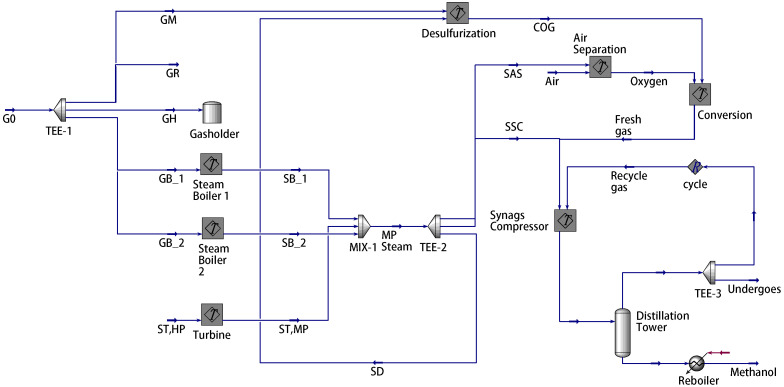
Gas-to-methanol process based on Aspen Hysys (blue arrow: material stream; red arrow: energy stream).

**Figure A1:**
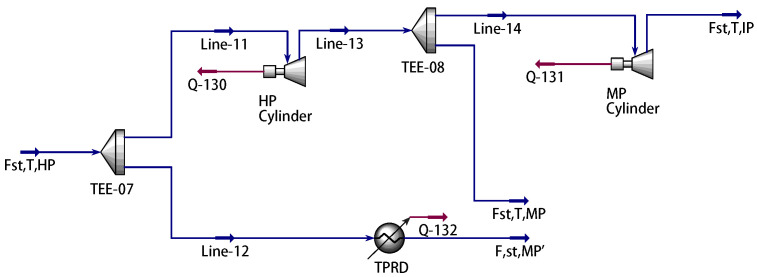
Steam turbine model based on Aspen Hysys (blue arrow: material stream; red arrow: energy stream).

**Figure A2:**
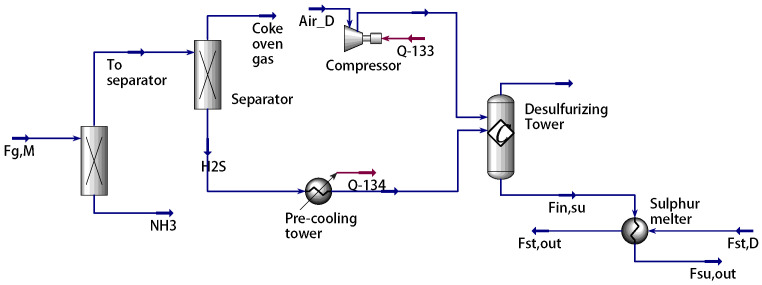
Desulfurization model based on Aspen Hysys (blue arrow: material stream; red arrow: energy stream).

**Figure A3:**
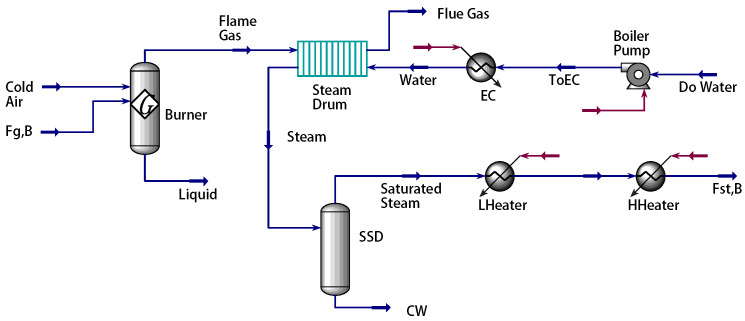
Steam boiler model based on Aspen Hysys (blue arrow: material stream; red arrow: energy stream).

**Figure A4:**
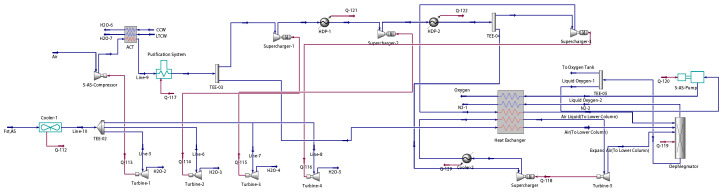
Air separation model based on Aspen Hysys (blue arrow: material stream; red arrow: energy stream).

**Figure A5:**
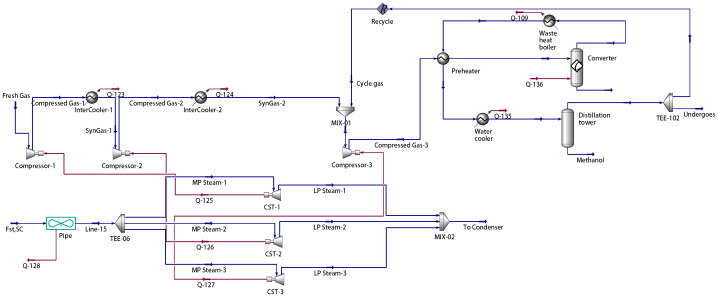
Syngas compressor model based on Aspen Hysys (blue arrow: material stream; red arrow: energy stream).

**Figure A6:**
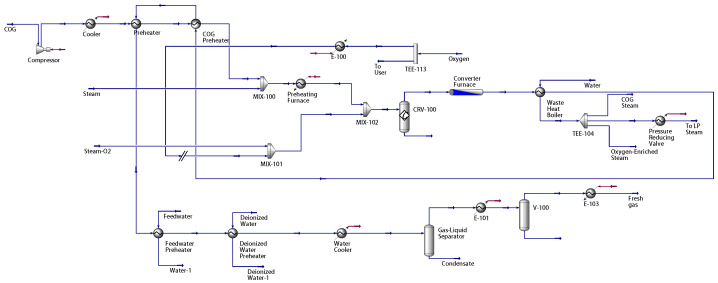
Conversion section model based on Aspen Hysys (blue arrow: material stream; red arrow: energy stream).

This section presents case studies based on a 250,000 t/a gas-to-methanol process at Shandong Province in China. A simulation model was developed using Aspen HYSYS V11, a widely used software in chemical industries, to simulate this process in Figure 3. Figure A1, Figure A2, Figure A3, Figure A4, Figure A5 Figure A6 depict Aspen models for the packaged subprocesses, including the steam boiler, desulfurization, air separation, and syngas compressor. Note that there are two identical steam boilers ($\lambda_{B}=2$). The process operation manual specifies the values and reference ranges for the parameters and variables in Table 2, Table 3, Table 4 and Table 5 [2,28].

**Figure 4:**
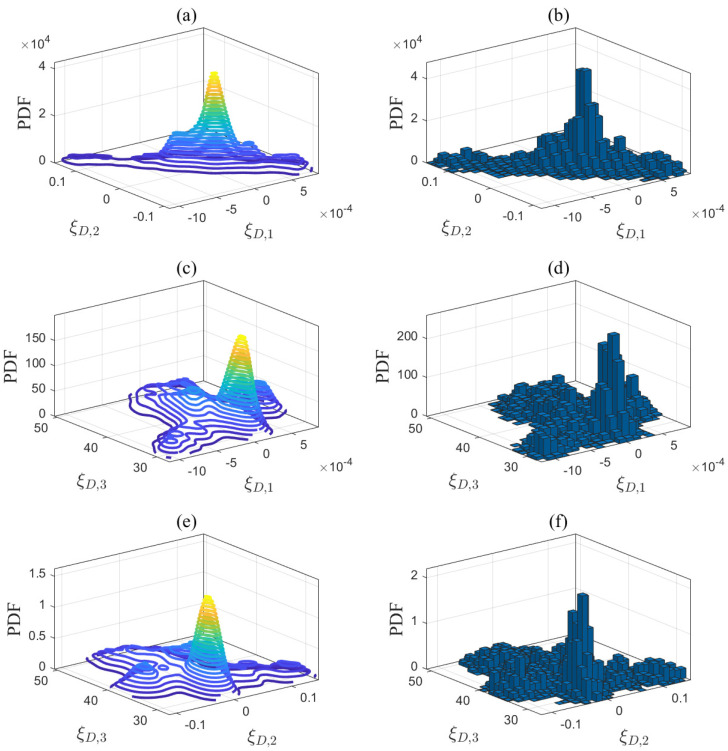
Projections of the posterior PDF in (**a**) $\xi_{D,1}$-$\xi_{D,2}$, (**c**) $\xi_{D,1}$-$\xi_{D,3}$, and (**e**) $\xi_{D,2}$-$\xi_{D,3}$, and MCMC sampling results in (**b**) $\xi_{D,1}$-$\xi_{D,2}$, (**d**) $\xi_{D,1}$-$\xi_{D,3}$, and (**f**) $\xi_{D,2}$-$\xi_{D,3}$ for the desulfurization model.

**Figure 5:**
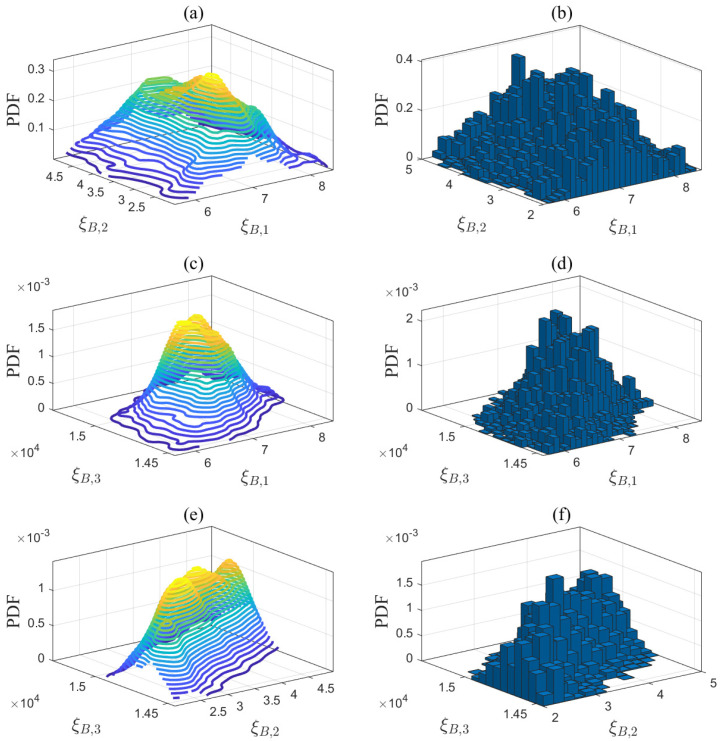
Projections of the posterior PDF in (**a**) $\xi_{B,1}$-$\xi_{B,2}$, (**c**) $\xi_{B,1}$-$\xi_{B,3}$, and (**e**) $\xi_{B,2}$-$\xi_{B,3}$, and MCMC sampling results in (**b**) $\xi_{B,1}$-$\xi_{B,2}$, (**d**) $\xi_{B,1}$-$\xi_{B,3}$, and (**f**) $\xi_{B,2}$-$\xi_{B,3}$ for the steam boiler model.

**Figure 6:**
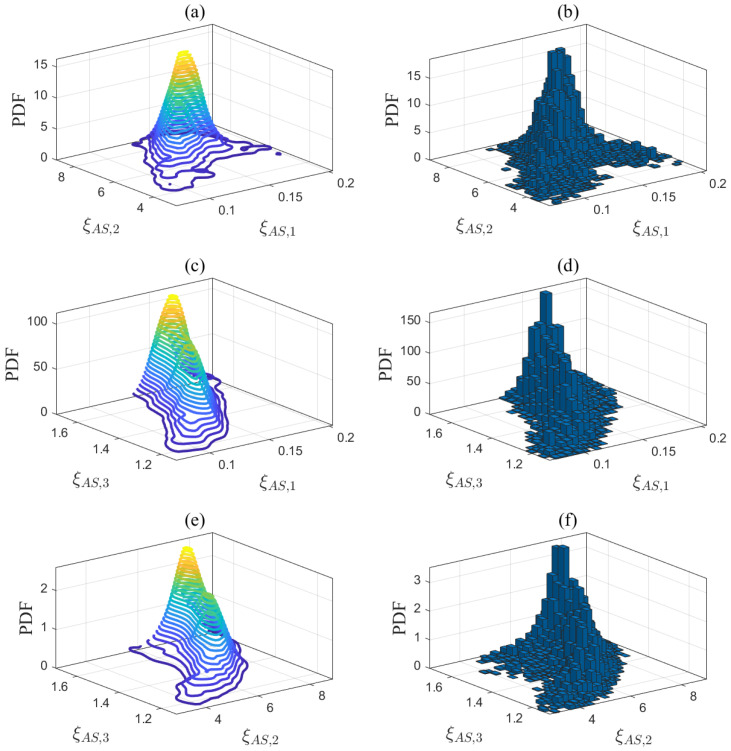
Projections of the posterior PDF in (**a**) $\xi_{AS,1}$-$\xi_{AS,2}$, (**c**) $\xi_{AS,1}$-$\xi_{AS,3}$, and (**e**) $\xi_{AS,2}$-$\xi_{AS,3}$, and MCMC sampling results in (**b**) $\xi_{AS,1}$-$\xi_{AS,2}$, (**d**) $\xi_{AS,1}$-$\xi_{AS,3}$, and (**f**) $\xi_{AS,2}$-$\xi_{AS,3}$ for the air separation model.

**Figure 7:**
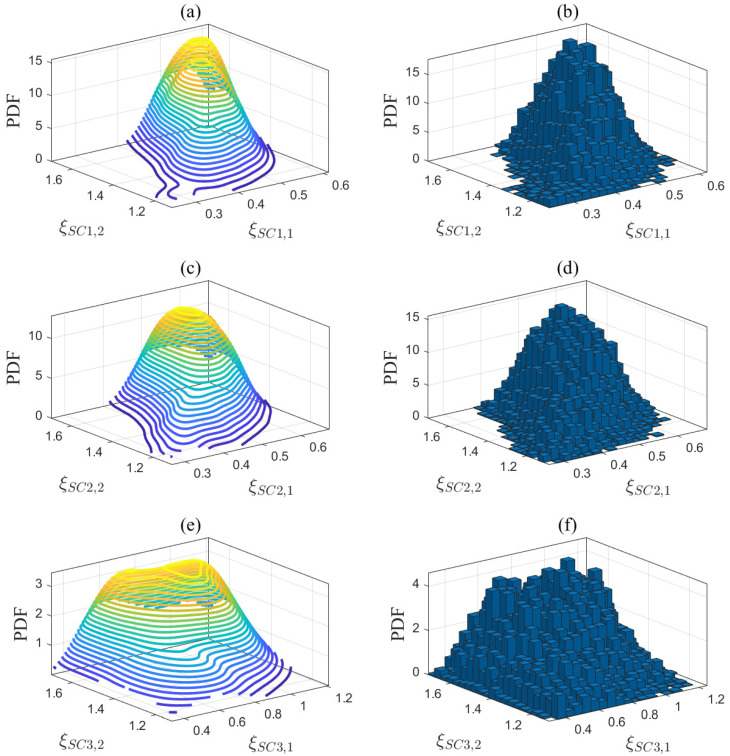
The posterior PDFs in (**a**) $\xi_{SC1,1}$-$\xi_{SC1,2}$, (**c**) $\xi_{SC2,1}$-$\xi_{SC2,2}$, and (**e**) $\xi_{SC3,1}$-$\xi_{SC3,2}$, and MCMC sampling results in (**b**) $\xi_{SC1,1}$-$\xi_{SC1,2}$, (**d**) $\xi_{SC2,1}$-$\xi_{SC2,2}$, and (**f**) $\xi_{SC3,1}$-$\xi_{SC3,2}$ for the syngas compressor model.

The first step of the proposed method is to estimate the unknown model parameters in (1)–(5). Let us take the desulfurization model in (1) as an example to explain Step 1. The prior parameter matrix $D_{D}^{\left(pr\right)}$ is estimated based on the historical data from Aspen Hysys. $D_{D}^{\left(pr\right)}$ is then applied to quantify the modeling uncertainties according to the Bayesian estimation theory outlined in Section 3.2.1. In (14), the input variable is the gas flow rate $F_{g,M}$, while the output variable is the steam consumption flow rate $F_{st,D}$. The operating conditions are defined by the inlet and outlet steam temperatures, which are denoted as $O_{D}=\left[T_{st,in},T_{st,out}\right]$. The step size for these operating conditions is set to 1 °C based on the sensor measurement resolution. According to the reference ranges in Table 2, namely, $T_{st,in}=400:1:450$ °C and $T_{st,out}=365:1:375\;$ °C, a total of 500 possible combinations ${\left\{O_{D,n}\right\}}_{n=1}^{500}$ of operating conditions are traversed. Using a ramp signal $F_{g,M}=50:0.1:80\;{\mathrm{km}}^{3}/h$ as the input data for the Aspen model in Figure A2, simulation experiments were conducted under 500 different operating conditions ${\left\{O_{D,n}\right\}}_{n=1}^{500}$. A total of 500 sets of historical data were generated as $\left\{{\hat{F}}_{st,x,n}\left(i\right);i=1,2,\ldots ,300;n=1,2,\ldots ,500\right\}$. For each set of historical data, the unknown parameters $\xi_{D,n}^{\left(pr\right)}=\left[\xi_{D,n1}^{\left(pr\right)},\xi_{D,n2}^{\left(pr\right)},\xi_{D,n3}^{\left(pr\right)}\right]$ of the desulfurization model were estimated in (15), resulting in $D_{D}^{\left(pr\right)}$. The same method was applied to the steam boiler, air separation, and syngas compressor models, generating $D_{B}^{\left(pr\right)}$, $D_{AS}^{\left(pr\right)}$, $D_{SC1}^{\left(pr\right)}$, $D_{SC2}^{\left(pr\right)}$, and $D_{SC3}^{\left(pr\right)}$. Table 6 presents the number of identified models and the average fitness, indicating that these models are highly accurate and suitable for subsequent optimization tasks. In addition, a new dataset was generated under a new set of operating conditions that were different from those of the identified models. The average fitness between this dataset and the identified models is presented in Table 6. The fitting results, all exceeding 90%, indicate that the established models exhibit generalization abilities. Each dimension of these parameter matrices was divided into equal intervals, and the posterior PDFs of the model parameters are quantified in (22) using Bayesian estimation theory. The quantification results are depicted by the contour plots shown in Figure 4, Figure 5, Figure 6 and Figure 7.

Second, one set of random parameter samples, as shown in Table 7, was drawn from the posterior PDFs in Figure 4, Figure 5, Figure 6 and Figure 7 using the MCMC method described in Section 3.2.2. These samples were used as the input data for the optimization problem in (36), and the optimization procedure in Steps 2 and 3 of the proposed method has been described in detail. The known conditions were as follows: the gas supply $F_{g,0}$ was 110 ${\mathrm{km}}^{3}/h$, the electricity demand $E_{dem}$ was 40 MW, and the initial volume $V_{H,0}$ of the gas holder was 13 ${\mathrm{km}}^{3}$. The energy purchase and selling prices provided in Table 8 were utilized, with the following parameter values set: the return gas ratio $\gamma_{R}$ was 0.45, the steam turbine extraction rate $\gamma_{ex}$ was 0.5, the gas storage loss rate $\gamma_{H,L}$ was 0.21, and the steam safety margin $\gamma_{S}$ was 0.1. The methanol synthesis reactor operates at 240 °C and 7.1 MPa, with the minimum methanol production threshold set at 18 t/h. The molar fractions of the gas components entering the reactor are as follows: ${\left(n_{CO}\right)}_{in}=9.51\%$, ${\left(n_{{CO}_{2}}\right)}_{in}=11.86\%$, ${\left(n_{H_{2}}\right)}_{in}=69.21\%$, ${\left(n_{H_{2}O}\right)}_{in}=0.12\%$, ${\left(n_{{CH}_{4}}\right)}_{in}=3.29\%$, ${\left(n_{N_{2}}\right)}_{in}=5.55\%$, and ${\left(n_{MeOH}\right)}_{in}=0.46\%$. A set of operating conditions, as shown in Table 9, was randomly generated from uniform distributions within the reference ranges specified in Table 2 to simulate the actual operating environment.

**Figure 8:**
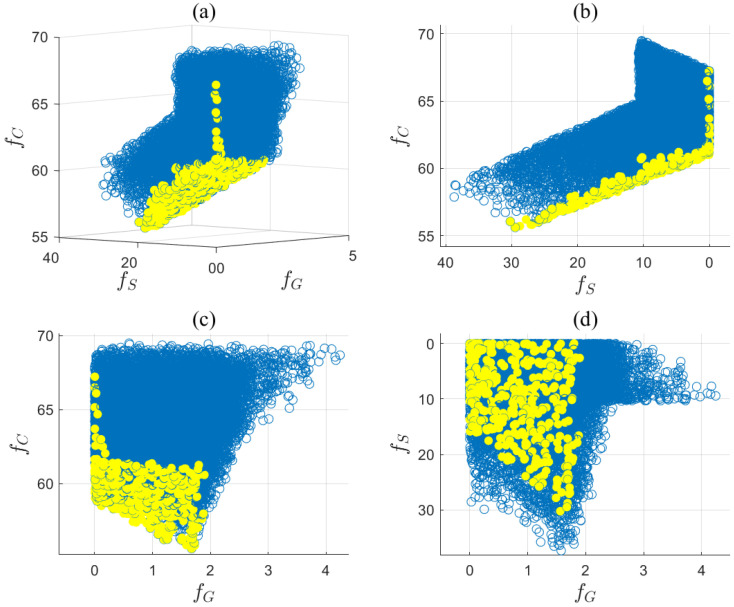
(**a**) Pareto front points (yellow) and set of feasible points (blue); (**b**) View of the $f_{S}$-$f_{C}$ plane; (**c**) View of the $f_{G}$-$f_{C}$ plane; (**d**) View of the $f_{G}$-$f_{S}$ plane.

**Figure 9:**
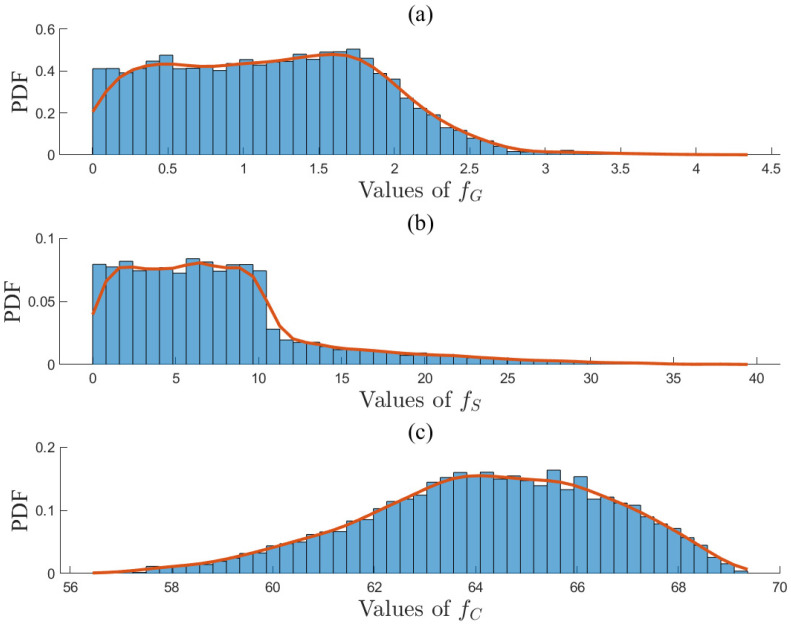
PDFs (red lines) of three objective functions (**a**) $f_{G}$, (**b**) $f_{S}$, and (**c**) $f_{C}$ on the Pareto Front.

**Figure 10:**
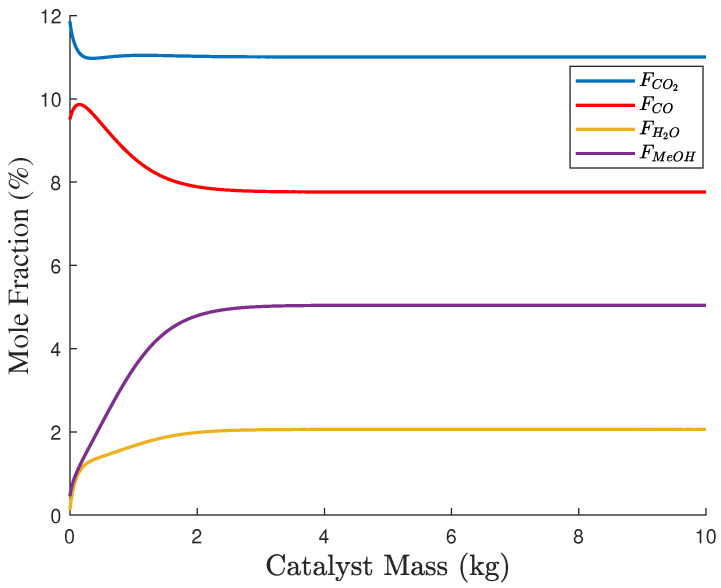
Concentration profiles in the methanol synthesis reactor.

The optimal solution set ${\left\{x_{p}\right\}}_{p=1}^{367}$ on the Pareto front was obtained by solving (36). The objective function values of $Y_{pq}$ in (40) based on ${\left\{x_{p}\right\}}_{p=1}^{367}$ are presented in a three-dimensional graph shown in Figure 8a. Here, the yellow points represent the Pareto front points, while the blue circles correspond to discrete data points within the feasible region. The projections on two-dimensional planes of these data points are shown in Figure 8b–d. The smoothness of the Pareto front surface fitted from the yellow points indicates that ${\left\{x_{p}\right\}}_{p=1}^{367}$ is close to the true solution set. The entropy values for the objective functions $f_{G}$, $f_{S}$, and $f_{C}$ were calculated by (41) and (42) as $H_{1}=0.945$, $H_{2}=0.949$, and $H_{3}=0.981$. Additionally, the PDFs of $f_{G}$, $f_{S}$, and $f_{C}$ are presented in Figure 9a,b, and c, respectively. The distributions of $f_{G}$ and $f_{S}$ are relatively concentrated, indicating small uncertainties, while the distribution of $f_{C}$ is more uniform, suggesting a larger uncertainty. Therefore, $f_{C}$ was down-weighted, resulting in entropy weights derived from Equation (43) as $\left(w_{1},w_{2},w_{3}\right)=\left(0.437,0.412,0.151\right)$. The ideal optimal solution in $Y_{pq}$ was evaluated based on Equation (44) and is given by $\left(F_{st,T}^{*}\left(1\right),F_{g,M}^{*}\left(1\right),F_{g,B}^{*}\left(1\right),F_{g,H}^{*}\left(1\right)\right)=\left(25.26,53.49,4.90,-2.86\right)$. If the entropy-weighted TOPSIS approach were to be applied in Step 3, the decision results in ${\left\{x_{p}\right\}}_{p=1}^{367}$ will be identical to those obtained using the entropy weight method. The optimal allocation of gas, steam, and electricity, along with the objective results, are presented in Table 10, while the concentration profiles in the methanol synthesis reactor are shown in Figure 10. The concentrations of key components at the reactor outlet are as follows: ${\left(n_{CO}\right)}_{out}=7.76\%$, ${\left(n_{{CO}_{2}}\right)}_{out}=11.01\%$, ${\left(n_{H_{2}O}\right)}_{out}=2.06\%$, and ${\left(n_{MeOH}\right)}_{out}=5.04\%$. The methanol production rate was 18.51 t/h, which complies with the relevant regulations in (35).

**Figure 11:**
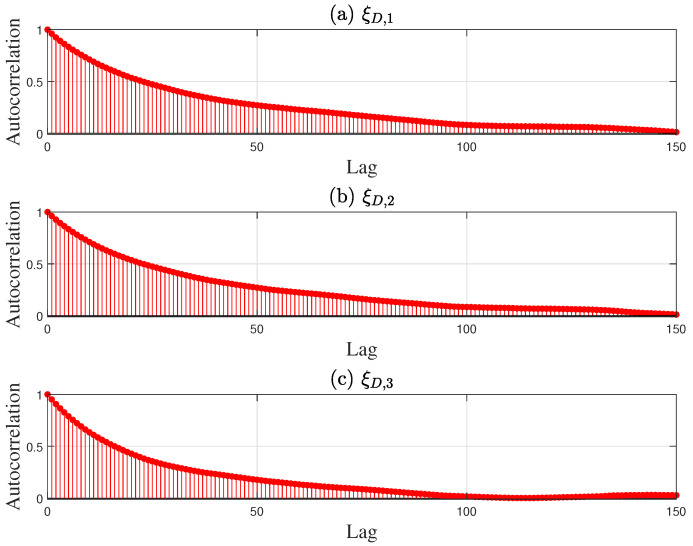
Sample autocorrelation of three unknown parameters (**a**) $\xi_{D,1}$, (**b**) $\xi_{D,2}$, and (**c**) $\xi_{D,3}$ for the desulfurization model.

Third, the parameter samples in Table 7 were updated without altering the known conditions and operating conditions to obtain the final solution in Steps 4 and 5 of the proposed method. As an example, random samples were drawn from the PDF of the desulfurization model in Figure 4 using the MCMC approach. The Markov chain for the desulfurization model converged after 150 sampling iterations in Figure 11, indicating that the current samples effectively simulate the posterior distribution. Based on the convergence of the Markov chains across all mechanistic models, 500 parameter samples were drawn, and the final sampling results are presented in the histograms in Figure 4, Figure 5, Figure 6 and Figure 7. A total of 500 ideal optimal solutions were obtained by repeating the solution procedure in Steps 2 and 3. The expected optimal values in (45) were then regarded as the final solution to the optimization problem in (36), specifically $\left({\bar{F}}_{st,T}^{*},{\bar{F}}_{g,M}^{*},{\bar{F}}_{g,B}^{*},{\bar{F}}_{g,H}^{*}\right)=\left(26.52,52.97,4.92,-2.32\right)$. If the sample average approximation approach was to be applied in Step 1, the resulting solution would be $\left(26.75,52.84,5.01,-2.35\right)$. The similarity of the results obtained by both approaches demonstrates the reliability of the proposed method. As a comparison, another experiment using a standard deterministic optimization was conducted. The operating conditions and unknown parameters were set to the nominal values shown in Table 2 and Table 7. These nominal values were used as the deterministic conditions for the optimization problem, and the corresponding deterministic solution was obtained as $F_{st,T}$ = 26.97 t/h, $F_{g,M}$ = 53.61 ${\mathrm{km}}^{3}/h$, $F_{g,B}$ = 3.89 ${\mathrm{km}}^{3}/h$, and $F_{g,H}$ = −2.61 ${\mathrm{km}}^{3}/h$.

**Figure 12:**
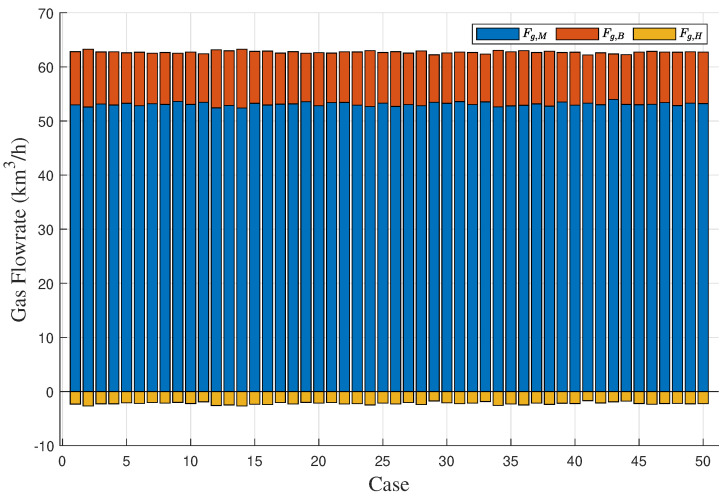
Allocation results of gas energy for 50 sets of cases.

**Figure 13:**
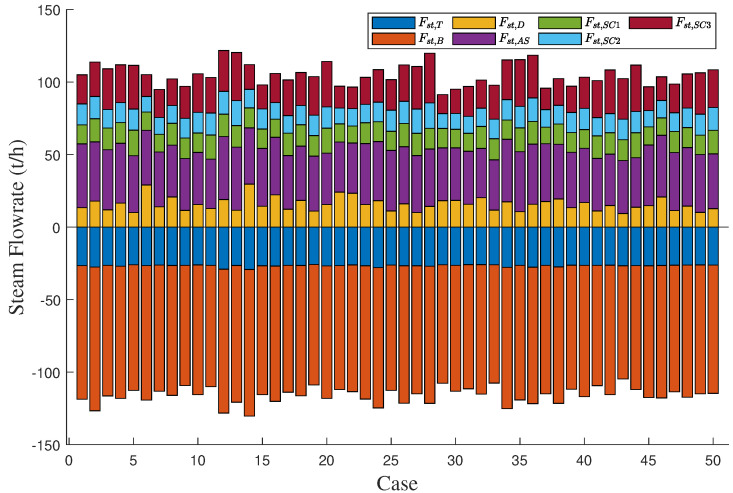
Allocation results of steam energy for 50 sets of cases.

**Figure 14:**
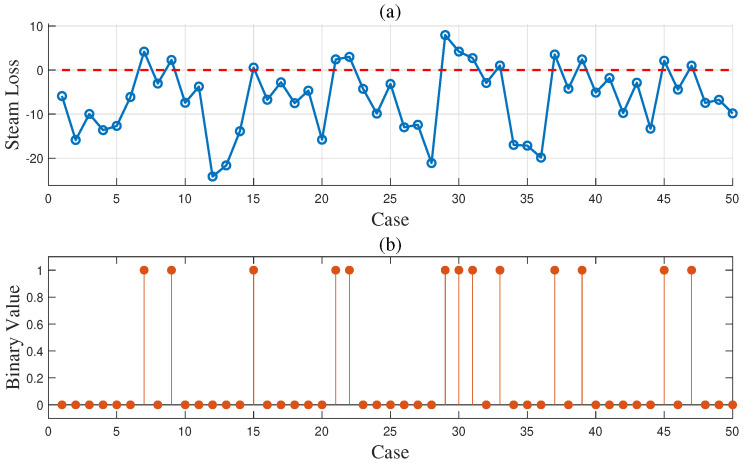
(**a**) Steam loss results (red dashed line: zero steam loss line) and (**b**) execution results of deterministic optimization for 50 sets of cases.

**Figure 15:**
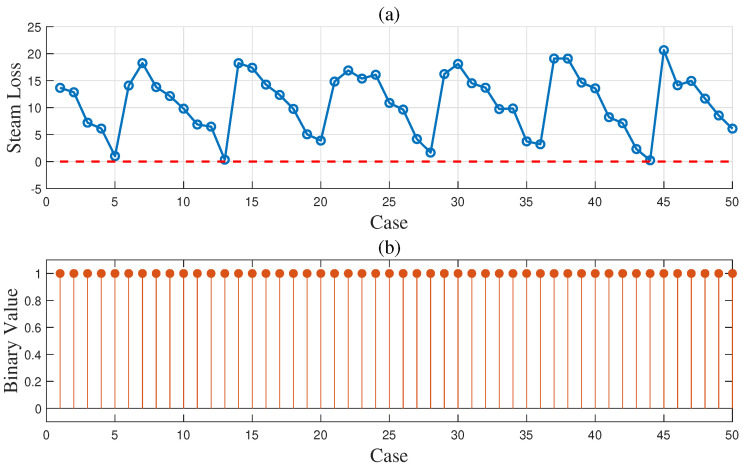
(**a**) Steam loss results (red dashed line: zero steam loss line) and (**b**) execution results of uncertainty optimization for 50 sets of cases.

To demonstrate the effectiveness of the proposed method in the gas-to-methanol process, the executability of both the deterministic and uncertain optimization solutions was analyzed. The solutions were sent into the Aspen model shown in Figure 3 to determine whether they can be implemented successfully. If the implementation is successful, it indicates that the steam supply is greater than or equal to the steam demand, thus satisfying the constraint in (27) so that the solution is confirmed to be executable. Conversely, if the implementation fails, it indicates that the solution is nonexecutable. A total of 50 cases were designed to verify the executability of the solutions. The above case described in details served as the first one, while the known conditions and unknown parameter samples for the remaining 49 cases were kept consistent with those of the first case. A total of 50 sets of random operating conditions were used to represent different scheduling scenarios for optimization. The optimization procedure for the first case was repeated analogously for 49 times to compute the solutions for the other 49 cases. The energy distribution results for gas and steam across the 50 cases are displayed in Figure 12 and Figure 13, respectively. In the deterministic optimization, the deterministic solution was applied to the 50 cases, and its executability is illustrated in Figure 14. The difference between the steam supply and steam demand across 50 sets of cases is presented in Figure 14a, where the red dashed line indicates the zero loss line for steam. Cases below this dashed line are considered nonexecutable. Figure 14b uses binary values to represent executability: executable cases are assigned a value of ‘1’, while nonexecutable cases are assigned a value of ‘0’. For the deterministic optimization, the validation results reveal that 37 sets of cases are nonexecutable, while only 13 sets are executable. This demonstrates that, under most operating conditions, deterministic optimization may produce results that are not executable, leading to outcomes that contradict the intended objectives. On the contrary, for the uncertainty optimization, the verification results in Figure 15 demonstrate that all optimization solutions in Figure 12 and Figure 13 are executable across all 50 sets of cases. This confirms the rationale and necessity of incorporating model uncertainties in the optimization of the gas-to-methanol process, resulting in a 74% improvement in executability compared to the deterministic optimization.

## 5. Conclusions

This paper proposed an entropy-based stochastic optimization method for a multi-energy system in a gas-to-methanol process, focusing on optimizing the allocation of gas, steam, and electricity to ensure executability under modeling uncertainties. The mechanistic models were developed at first for chemical equipments, including the desulfurization, air separation, syngas compressors, and steam boilers, whose parameters were estimated based on historical data. Structural errors in these mechanistic models under varying operating conditions resulted in noticeable model uncertainties. Bayesian estimation theory was used to fit probability density functions (PDFs) of the estimated model parameters by analyzing the differences between historical data and model predictions under varying operating conditions. The Markov Chain Monte Carlo approach was applied to generate parameter samples from these PDFs, decomposing the uncertain optimization problem into multiple deterministic ones. The optimization problem aimed to minimize gas loss, steam loss, and operating costs under constraints related to model uncertainties, equipment capacities, and energy balance. In the multi-objective optimization, the Pareto front solution set was filtered using the entropy weight approach, selecting a final solution with the minimal subjectivity and preferences. Case studies with Aspen Hysys-based simulations showed that optimization solutions considering model uncertainties improved executability by 74% compared to the counterparts from a standard deterministic optimization.

We are currently applying the optimization solutions obtained in this paper to a real industrial setting. The chemical company employs a manual, experience-based scheduling method that leads to significant waste of medium-pressure steam. Therefore, the application of the proposed optimization method is expected to yield satisfactory improvements in the company’s energy efficiency and economic performance. 

## Figures and Tables

**Table 1:** Parameter values of the steady-state kinetic models.

Parameter	$A_{i}$	$B_{i}$
$k_{1}$	1.07	−94,765
$k_{2}$	3453.38	0
$k_{3}$	0.499	17,197
$k_{4}$	$6.62\times 10^{-11}$	124,119
$k_{5}$	$1.22\times 10^{10}$	−94,765

**Table 2:** Reference ranges and nominal values of operating conditions.

Model	Operating Condition	Reference Range	Nominal Value	Operating Condition	Reference Range	Nominal Value
D	$T_{st,in}$	400–450 °C	436 °C	$T_{st,out}$	365–375 °C	370 °C
B	$T_{EG}$	85–135 °C	120 °C	$T_{CA}$	20–30 °C	25 °C
AS	$T_{AS,in,1}$	5–40 °C	20 °C	$\eta_{T}$	70–90%	75%
SC1	$T_{SC1,out}$	85–105 °C	88 °C	$\eta_{T1}$	70–90%	75%
SC2	$T_{SC2,out}$	85–105 °C	85 °C	$\eta_{T2}$	70–90%	75%
SC3	$T_{SC3,out}$	45–60 °C	48 °C	$\eta_{T3}$	70–90%	75%

**Table 3:** Known values of the parameters.

Known Parameter	Value	Known Parameter	Value	Known Parameter	Value
$T_{su,in}$ (°C)	29.81	$T_{su,out}$ (°C)	83.46	$H_{S}$ (kJ/kg)	3312
$H_{FW}$ (kJ/kg)	443	$P_{AS,in,1}$ (MPa)	0.1	$P_{AS,out,1}$ (MPa)	0.5
$P_{AS,in,2}$ (MPa)	0.5	$P_{AS,out,2}$ (MPa)	4	$T_{AS,in,2}$ (°C)	11
$T_{T,in}$ (°C)	436	$P_{T,in}$ (MPa)	3.43	$P_{T,out}$ (MPa)	0.07
$P_{SC1,in}$ (MPa)	2.1	$P_{SC1,out}$ (MPa)	3.47	$T_{SC1,in}$ (°C)	40
$P_{SC2,in}$ (MPa)	3.47	$P_{SC2,out}$ (MPa)	5.5	$T_{SC2,in}$ (°C)	40
$P_{SC3,in}$ (MPa)	5.5	$P_{SC3,out}$ (MPa)	6	$T_{SC3,in}$ (°C)	40
*R* (kJ/(kg·K))	0.29	$R_{u}$ (J/(mol·K))	8.31	$M_{1}\left(g/\mathrm{mol}\right)$	11.23
$M_{2}$ (g/mol)	11.23	$M_{3}$ (g/mol)	10.17	$\gamma_{f}$	1.42
$C_{SC,1}$	0.12	$C_{SC,2}$	−11.34	$C_{SC,3}$	661.04
$\eta_{ST}$ (%)	75	$H_{ST,HP}$ (kJ/kg)	212.7	$H_{ST,MP}$ (kJ/kg)	1167.9
$\rho_{c}$ ($\mathrm{kg}/m^{3}$)	1750	$D_{p}$ (mm)	10	∅ (%)	50
W (kg)	10	$A_{c}$ ($m^{2}$)	5	μ (Pa·s)	$1.5\times 10^{-5}$

**Table 4:** Reference ranges of key variables.

Key Variable	Reference Range	Key Variable	Reference Range
$F_{g,0}$ (${\mathrm{km}}^{3}/h$)	100–120	$F_{g,M}$ (${\mathrm{km}}^{3}/h$)	50–80
$F_{g,B}$ (${\mathrm{km}}^{3}/h$)	3.3–13.3	$F_{g,H}$ (${\mathrm{km}}^{3}/h$)	−3–3
$V_{H}$ (${\mathrm{km}}^{3}$)	0–20	$F_{st,D}$ (t/h)	≤20
$F_{st,AS}$ (t/h)	40–55	$F_{st,SC}$ (t/h)	30–50
$F_{st,T}$ (t/h)	10–40	$F_{st,B}$ (t/h)	≤75
$E_{ST}$ (MW)	≤15	$E_{dem}$ (MW)	40–50

**Table 5:** Reference ranges of the parameters.

Parameter	Reference Range	Parameter	Reference Range
$C_{su}$ (kJ/(kg·°C))	0.71–1.09	$C_{st}$ (kJ/(kg·°C))	1.5–2.5
$k_{B,1}$	0.2–1.7	$k_{B,2}$	3.55–3.9
$\alpha_{EG}$	1.5–1.8	$q_{L}$ (%)	2–8
$Q_{B}$ (kJ/kg)	8400–33,500	$\rho_{A,1}$, $\rho_{A,2}$ ($\mathrm{kg}/m^{3}$)	1.0–1.4
$\eta_{I,1}$, $\eta_{I,2}$ (%)	70–85	$\rho_{1}$, $\rho_{2}$, $\rho_{3}$	0.4–0.9
$\eta_{P,1}$, $\eta_{P,2}$, $\eta_{P,3}$ (%)	70–84	$k_{T}$, $k_{T1}$, $k_{T2}$, $k_{T3}$	1.1–1.7

**Table 6:** Number of identified models and average fitness.

Model	Number of Models	Average Fitness	Generalization Ability
Steam Boiler	500	93.50%	91.27%
Desulfurization	500	92.94%	90.53%
Air Separation	756	99.78%	98.25%
Syngas Compressor Stage 1	378	99.99%	98.39%
Syngas Compressor Stage 2	462	99.99%	98.57%
Syngas Compressor Stage 3	273	99.47%	97.15%

**Table 7:** Unknown parameter samples based on the MCMC approach and their corresponding nominal values.

Unknown Parameter	Sampled Value	Nominal Value	Unknown Parameter	Sampled Value	Nominal Value	Unknown Parameter	Sampled Value	Nominal Value
$\xi_{D,1}$	1.72 × 10^−5^	−9.56 × 10^−4^	$\xi_{D,2}$	−0.0037	0.12	$\xi_{D,3}$	36.66	29.36
$\xi_{B,1}$	14.28	11.06	$\xi_{B,2}$	7.10	6.58	$\xi_{B,3}$	29,755.78	29,207.32
$\xi_{AS,1}$	0.15	0.15	$\xi_{AS,2}$	6.74	7.47	$\xi_{AS,3}$	1.50	1.61
$\xi_{SC1,1}$	0.49	0.46	$\xi_{SC1,2}$	1.49	1.62	$\xi_{SC2,1}$	0.52	0.47
$\xi_{SC2,2}$	1.47	1.65	$\xi_{SC3,1}$	0.78	0.60	$\xi_{SC3,2}$	1.49	1.63

**Table 8:** Purchase and selling prices of steam, gas, and electricity.

Item	Value
Medium-pressure steam selling price $c_{st,MP}$	190 CNY/kg
High-pressure steam purchase price $c_{st,HP}$	234 CNY/kg
Electricity selling price in the grid $c_{grid}$	490 CNY/kW
Coke oven gas purchase price $c_{g}$	500 $\mathrm{CNY}/m^{3}$
Self-generated electricity price $c_{ST}$	220 CNY/kW

**Table 9:** Random values of operating conditions from uniform distributions.

Operating Condition	Random Value	Operating Condition	Random Value
$T_{st,in}$	430.37 °C	$T_{st,out}$	370.46 °C
$T_{EG}$	122.62 °C	$T_{CA}$	26.17 °C
$T_{AS,in,1}$	29.63 °C	$\eta_{T}$	70.32%
$T_{SC1,out}$	100.86 °C	$\eta_{T1}$	83.98%
$T_{SC2,out}$	96.38 °C	$\eta_{T2}$	72.73%
$T_{SC3,out}$	55.41 °C	$\eta_{T3}$	86.99%

**Table 10:** Energy allocation and objective results after optimization.

Symbol	After Optimization	Symbol	After Optimization
$F_{st,T}$ (t/h)	25.26	$F_{g,M}$ (${\mathrm{km}}^{3}/h$)	53.49
$F_{g,B}$ (${\mathrm{km}}^{3}/h$)	4.90	$F_{g,H}$ (${\mathrm{km}}^{3}/h$)	−2.86
$V_{H}$ (${\mathrm{km}}^{3}$)	8.38	$F_{st,D}$ (t/h)	12.74
$F_{st,AS}$ (t/h)	43.06	$F_{st,SC}$ (t/h)	48.77
$F_{st,B}$ (t/h)	45.61	$E_{ST}$ (MW)	8.38
$E_{grid}$ (MW)	31.62	$f_{G}$ (${\mathrm{km}}^{3}/h$)	0.07
$f_{S}$ (t/h)	0.262	$f_{C}$ (CNY)	61,341.1

## Data Availability

All data are presented in the main text.

## Nomenclature

$\alpha_{EG}$	excess air coefficient for exhaust gas
∅	porosity of the catalyst
$\eta_{I,1}$, $\eta_{I,2}$	isentropic efficiency of air separation turbines
$\eta_{P,l}$	polytropic efficiency of *l*-th stage of syngas compressor
$\eta_{ST}$	isentropic efficiency of steam turbine
$\eta_{Tl}$	isentropic efficiency of *l*-th stage of syngas compressor turbine
$\eta_{T}$	isentropic efficiency of air separation turbine
$\gamma_{R}$	return ratio of gas supply back to the coking combustor
$\gamma_{S}$	steam safety margin
$\gamma_{ex}$	extraction rate of steam drawn from the high-pressure cylinder
$\gamma_{H,L}$	loss rate of gas storage
$\lambda_{B}$	the number of steam boilers in the energy system
μ	viscosity of gas passing through the bed
$\rho_{c}$	solid density of the catalyst
$\rho_{A,1}$, $\rho_{A,2}$	inlet gas density of air separation compressors
$\rho_{l}$	fresh gas inlet relative density of *l*-th stage of syngas compressor
$A_{c}$	cross-sectional area of the reactor
$C_{D,1}$	polynomial parameters of desulfurization model
$C_{D,2}$	polynomial parameters of desulfurization model
$C_{D,3}$	polynomial parameters of desulfurization model
$C_{st}$	specific heat capacity of steam at constant pressure
$C_{su}$	specific heat capacity of sulfur at constant pressure
$D_{p}$	diameter of particle in the bed
$H_{S}$	enthalpy value of steam
$H_{FW}$	enthalpy value of feed water
$H_{ST,r}$	steam entropy difference between the inlet and outlet of the
	low-pressure (high-pressure) cylinder
$k_{B,1}$	calculation coefficient
$k_{B,2}$	calculation coefficient
$k_{Tl}$	steam adiabatic index of *l*-th stage of syngas compressor turbine
$k_{T}$	steam adiabatic index of air separation turbine
$M_{l}$	molar mass of inlet gas at *l*-th stage of syngas compressor
$Q_{B}$	total heat value of fuel
$q_{L}$	loss due to incomplete chemical combustion
*R*	gas constant for air
$R_{u}$	universal gas constant
*W*	mass of the catalyst
$E_{dem}$	electricity demand of the energy system
$E_{grid}$	purchased electricity from the grid
$E_{ST}$	self-generated electricity of the steam turbine
$F_{g,0}$	gas supply flow rate of the energy system
$F_{g,B}$	gas combustion flow rate in the steam boiler
$F_{g,H}$	gas throughput of the gas holder
$F_{g,M}$	gas flow rate into the methanol production line
$F_{g,R}$	return flow rate of gas in the coking combustor
$F_{st,AS}$	steam consumption flow rate for the air separation
$F_{st,B}$	steam production flow rate of the steam boiler
$F_{st,D}$	steam consumption flow rate for the desulfurization
$F_{st,SC}$	steam consumption flow rate for the syngas compressor
$F_{st,T}$	extraction steam flow rate of the steam turbine
$P_{AS,in,1}$, $P_{AS,in,2}$	air inlet pressure of air separation compressors
$P_{AS,out,1}$, $P_{AS,out,2}$	air outlet pressure of air separation compressors
$P_{SCl,in}$	inlet pressure of *l*-th stage of syngas compressor
$P_{SCl,out}$	outlet pressure of *l*-th stage of syngas compressor
$P_{T,in}$	steam inlet pressure of turbine
$P_{T,out}$	steam outlet pressure of turbine
$T_{AS,in,1}$, $T_{AS,in,2}$	air inlet temperature of air separation compressors
$T_{CA}$	cold air temperature
$T_{EG}$	exhaust gas temperature
$T_{SCl,in}$	inlet temperature of *l*-th stage of syngas compressor
$T_{SCl,out}$	outlet temperature of *l*-th stage of syngas compressor
$T_{st,in}$	inlet temperature of steam
$T_{st,out}$	outlet temperature of steam
$T_{su,in}$	inlet temperature of sulfur
$T_{su,out}$	outlet temperature of sulfur
$T_{T,in}$	steam inlet temperature of turbine
$V_{H}$	storage volume of the gas holder

## Appendix A

The encapsulated subprocesses of the Aspen model for the gas-to-methanol process described in Figure 3 include the steam boiler, desulfurization, air separation, and syngas compressor, as depicted below:
